# Ecological Perspectives on Microbes Involved in N-Cycling

**DOI:** 10.1264/jsme2.ME13159

**Published:** 2014-03-13

**Authors:** Kazuo Isobe, Nobuhito Ohte

**Affiliations:** 1 Department of Applied Biological Chemistry, Graduate School of Agricultural and Life Sciences, The University of Tokyo, 1–1–1 Yayoi, Bunkyo-ku, Tokyo 113–8657, Japan; 2 Department of Forest Science, Graduate School of Agricultural and Life Sciences, The University of Tokyo

**Keywords:** nitrogen cycle, ecosystem functions, microbial community dynamics

## Abstract

Nitrogen (N) cycles have been directly linked to the functional stability of ecosystems because N is an essential element for life. Furthermore, the supply of N to organisms regulates primary productivity in many natural ecosystems. Microbial communities have been shown to significantly contribute to N cycles because many N-cycling processes are microbially mediated. Only particular groups of microbes were implicated in N-cycling processes, such as nitrogen fixation, nitrification, and denitrification, until a few decades ago. However, recent advances in high-throughput sequencing technologies and sophisticated isolation techniques have enabled microbiologists to discover that N-cycling microbes are unexpectedly diverse in their functions and phylogenies. Therefore, elucidating the link between biogeochemical N-cycling processes and microbial community dynamics can provide a more mechanistic understanding of N cycles than the direct observation of N dynamics. In this review, we summarized recent findings that characterized the microbes governing novel N-cycling processes. We also discussed the ecological role of N-cycling microbial community dynamics, which is essential for advancing our understanding of the functional stability of ecosystems.

The fundamental issues of ecosystem ecology are related to understanding how ecosystems maintain functional stability and predicting how ecosystems respond to environmental changes. An ecosystem can be defined as an interacting system composed of an environment and all the organisms involved in it. Many ecosystem ecologists have focused on the N cycle or the dynamics of N transformation in various ecosystems because N (along with H, C, O, S and P), as a major component of proteins and nucleic acids, is an essential element for life and its supply can limit primary productivity in many natural terrestrial and marine ecosystems (12, 122). The N cycle has also been the focus of debate in nitrogen-rich ecosystems, such as fertilized agricultural fields or eutrophic rivers and coasts that are affected by anthropogenic N input (48, 53, 85, 90, 101, 110, 114, 115, 134, 135). Wastewater treatment systems are also examples of artificial ecosystems in which N removal is frequently studied (29, 78, 116, 121). Studies on the pathways and rates of input, output, and internal cycle of N and its interactions with other elements can provide insights into the fundamental issues related to ecosystem ecology.

Nitrogen is a versatile element that forms compounds in various oxidation states, ranging from −3 (ammonium and amino-nitrogen) to +5 (nitrate) (Fig. 1). N transitions between compounds with different oxidation states are largely driven by thermodynamically constrained redox reactions and are typically catalyzed by microbes (24). Given the ubiquity and biogeochemical contributions of microbes, microbial community dynamics may be directly associated with temporal and spatial variations in internal N-cycling pathways and rates in ecosystems. Microbiologists have already demonstrated the critical ecological roles that microbes play in N-cycling pathways and rates by integrating microbial community dynamics into N biogeochemical phenomena. Microbial community dynamics may also ultimately affect the functional stability of ecosystems. Ecosystem ecologists have frequently reported non-linear alterations in N dynamics and sometimes identified the thresholds at which these alterations occurred as ecosystems responded to perturbations or disturbances. For example, Aber *et al.* (1) proposed a conceptual model of N saturation in temperate forests in which the response of the forest to chronic atmospheric N deposition could be quantitatively classified into three progressive stages. The first stage was characterized by an increase in net soil N mineralization and tree growth. Although net soil N mineralization decreased in the second stage, nitrification was induced, resulting in more NO_3_^−^ leaching. Finally, the uptake of N by plants and tree growth declined, whereas nitrification and NO_3_^−^ loss continued to increase. Non-linear alterations in the N dynamics of ecosystems may be largely due to the non-linear responses of microbial community dynamics or the physiological constraints of the community. Therefore, ecosystem ecologists are beginning to explicitly consider the ecological roles of microbial community dynamics (2, 95). Few studies have investigated the ecological roles of an entire microbial community’s dynamics in the functional stability of the ecosystem, and this has been attributed to microbiologists being more inclined to focus on functionally equivalent microbial groups that are involved in specific (single or a few) N-cycling processes. However, ecological studies by microbiologists may provide valuable insights into the mechanisms of not only the N cycle of the ecosystem, but also its functional stability, and can ultimately permit predictions of the functional stability in a changing environment with an unprecedented level of detail.

We described recent findings on microbes governing novel N transformations in this brief review. We also discussed the relationship between microbial community dynamics and N biogeochemistry as well as the ecological roles of microbial community dynamics in the N-cycling rates and processes in ecosystems. We lastly highlighted pressing topics in microbiology that may advance our understanding of the role of microbial community dynamics in the functional stability of the ecosystem with a focus on N dynamics.

## New pathways and players

**Fig. 1:**
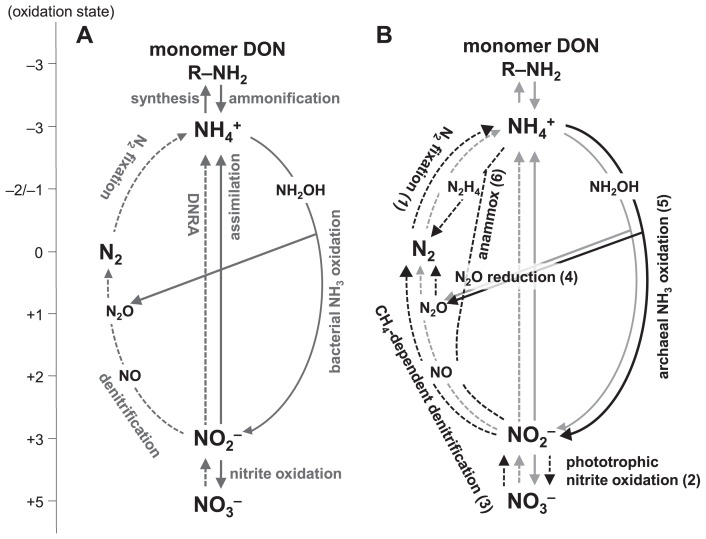
Schematic representation highlighting the main processes in the microbial N cycle, with a focus on (A, *gray*) the classical processes and (B, *black*) recently discovered processes discussed in the text. Processes mainly occurring under oxic and anoxic conditions are shown as solid and dashed arrows, respectively. Detailed reactions of recently discovered processes are described in Table 1. Note: denitrification (A), denitrification coupled to the oxidation of organic matter, hydrogen, reduced iron, or reduced sulfur species; nitrite oxidation (A), aerobic chemolithotrophic nitrite oxidation; N_2_ fixation(B), N_2_ fixation by unicellular cyanobacteria; N_2_O reduction (B), N_2_O reduction by non-denitrifying bacteria. Abbreviations: DON, dissolved organic N; DNRA, dissimilatory nitrate reduction to ammonia.

The primary ecological function of the N cycle is to provide N to organisms such as microbes, plants, and animals (118). Almost all prokaryotes (except for nitrogen-fixing bacteria and archaea) and eukaryotes require fixed forms of N (such as ammonium, nitrate, and monomer-dissolved N, which include amino acids and amino sugars) for their growth (Fig. 1). The physiology of the dissimilatory oxidative and reductive reactions involved in N-cycling has been studied extensively with isolated strains; however, these isolates have mainly been limited to N-fixing bacteria (*e.g.*, the genera *Azotobacter* and *Bradyrhizobium*), ammonia- oxidizing bacteria (AOB; *e.g.*, the genera *Nitrosomonas* and *Nitrosospira*), nitrite-oxidizing bacteria (NOB; *e.g.*, the genera *Nitrobacter* and *Nitrospira*), and heterotrophic-denitrifying bacteria (*e.g.*, the genera *Pseudomonas* and *Azospirillum*) (29, 41, 42, 59, 64, 91, 121). In addition to these culture-dependent studies (20, 88), molecular analyses (such as clone libraries and qPCR) have been performed with 16S rRNA genes (28, 48) as well as the “functional” genes involved in N-cycling: *nifH* (nitrogenase gene subunit H), *amoA* (ammonia monooxygenase gene subunit A), *nirS* and *nirK* (heme cd1-containing and copper-containing nitrite reductase genes), and *nosZ* (nitrous oxide reductase gene subunit Z) (39, 42, 43, 46, 70, 79, 91, 130). These studies have increased our understanding of the physiology and population dynamics of N_2_-fixers, AOB, NOB, and denitrifiers in both natural and artificial environments. Many microbes have also been identified as key players in novel N-cycling processes in the past few decades. In this section, we discussed recent studies on N-cycling microbes and their ecological functions from a biogeochemical perspective.

### 1) Unicellular N_2_ fixing cyanobacteria (Fig. 1B [1], Table 1)

Biological N_2_ fixation is the main process that controls the supply of N to organisms in the ocean (137). The filamentous nonheterocystous cyanobacteria of the genus *Trichodesmium* were believed to be the principal N_2_ fixers and suppliers of nitrogen compounds in oceanic N-cycling (13). They were also shown to be capable of CO_2_ fixation via the oxygenic photosynthetic pathway. However, the oxygen (O_2_) generated by these bacteria may inhibit the activity of nitrogenase, which is the key enzyme in N_2_ fixation, and the reason for this has yet to be determined (8). In an attempt to resolve this issue, Finzi-Hart *et al.* (27) analyzed the quantitative metabolic uptake patterns of NaH^13^CO_3_ and ^15^N_2_ in individual *Trichodesmium* cells using nanometer-scale secondary ion mass spectrometry (NanoSIMS). They found that the segregation of CO_2_ and N_2_ fixation in *Trichodesmium* was regulated in part by temporal factors.

In addition to *Trichodesmium* populations, two unicellular diazotrophic cyanobacteria (UCYN groups A and B) have been identified as major N-fixers in oceans (14, 15, 83). Although UCYN-A-type organisms have yet to be cultivated, Zehr’s group screened UCYN-A-type organisms using flow cytometry combined with a UCYN-A-specific qPCR assay and revealed that they did not have an oxygen-evolving photosystem II, RubisCo, or tricarboxylic acid (TCA) cycle in UCYN-A-type organism through a metagenomic analysis of the UCYN-A genomes (120, 138). Thompson *et al.* subsequently analyzed the quantitative metabolic uptake of NaH^13^CO_3_ and ^15^N_2_ in cells that were sorted by flow cytometry using halogenated *in situ* hybridization NanoSIMS (HISH-SIMS), and demonstrated that the UCYN-A-type organism formed a symbiotic partnership with a prymnesiophyte to maintain its growth. In this partnership, the UCYN-A-type organism received fixed carbon in exchange for fixed N, which was transferred to the prymnesiophyte (119). A previous study reported that *Crocosphaera watsonii* of the UCYN-B group possessed typical cyanobacterial photosynthetic machinery and its N_2_ fixation rates were the highest at night (137). These filamentous and unicellular diazotrophic cyanobacteria phylotypes are found in oligotrophic tropical oceans, and N_2_ fixation by UCYN-A/B-type cyanobacteria has been shown to account for a large fraction (>50%) of total N_2_ fixation in some locations (83, 139, 140).

Farnelid *et al.* (25) analyzed *nifH* genes in seawater samples collected from 10 different geographic locations using 454 pyrotag sequencing, and demonstrated that *nifH* gene clusters related to *Alpha*-, *Beta*-, and *Gamma-proteobacteria* were the most common and had distinct geographic distributions. These non-cyanobacteria groups may also play significant roles in global N-cycling (50). Thus, both cyanobacteria and non-cyanobacteria N-fixers are broadly distributed in marine environments, have unique ecophysiological traits, and may strongly influence the marine N budget (82, 84).

### 2) Phototrophic and unrealized chemolithotrophic nitrite-oxidizing bacteria (Fig. 1B [2], Table 1)

Nitrite functions as a substrate or intermediate in many N transformation processes, but does not generally accumulate in natural ecosystems (47). Nitrite produced via ammonia oxidation is readily oxidized to nitrate under oxic conditions. Therefore, the phylogeny, physiology, and ecological niches of nitrite-oxidizing bacteria are thought to be diverse. Only five genera of aerobic chemolithotrophic nitrite had been described until recently: *Nitrobacter*, *Nitrotoga*, and *Nitrococcus* within the *Alpha*-, *Beta*-, and *Gamma-proteobacteria*, respectively, and *Nitrospira* within the *Nitrospirae* and *Nitrospina* (66). The genus *Nitrospina* was provisionally assigned to the *Deltaproteobacteria* based on its 16S rRNA-based phylogenetic inference (107, 117); however, based on detailed phylogenetic analyses using concatenated marker genes in the *Nitrospina gracilis* genome, Lücker *et al.* (73) suggested that *Nitrospina* may form a novel bacterial phylum distinct from the *Proteobacteria*, and proposed the name *Nitrospinae*.

Recent findings have expanded the known physiology and phylogeny of nitrite oxidizers. Griffin *et al.* (33) enriched phototrophic nitrite oxidizers from freshwater sediments and sewage that could use nitrite as an electron donor for anoxygenic photosynthesis and stoichiometrically oxidized nitrite to nitrate. Two phototrophic nitrite-oxidizing strains, namely *Rhodopseudomonas* sp. strain LQ17 and *Thiocapsa* sp. strain KS1 within the *Alpha*- and *Gamma-proteobacteria*, respectively, were isolated from sewage (108). Although phototrophs generally have direct impacts on the N cycle through reductive processes such as nitrogen fixation, assimilation, and respiration, this discovery demonstrated that oxidation in N-cycling can be driven by photosynthesis. The numbers of these nitrite-oxidizing phototrophs were low in the most-probable-number (MPN) dilution assay, and the two isolates could use many reductants other than nitrite (organic compounds, H_2_, HS^−^, and Fe^2+^) as electron donors (108). These findings suggest that their functional importance in nitrite oxidation in natural environments may be limited. Sorokin *et al.* (111) isolated a chemolithotrophic nitrite oxidizer (*Nitrolancetus hollandicus*) belonging to the widespread phylum *Chloroflexi* from a bioreactor. *N. hollandicus* tolerates a broad temperature range (25–63°C) and high nitrite concentration (75 mM, half saturation constant Ks=1 mM) and can grow mixotrophically on nitrite and formate, which distinguishes it from all other known nitrite oxidizers. However, because most conventional nitrifying wastewater treatment plants are operated at lower temperatures and lower nitrite concentrations than optimal conditions for the growth of *N. hollandicus*, it is unlikely to contribute to nitrite oxidation during the treatment of wastewater. Although the functional importance of both nitrite-oxidizing phototrophs and chemolithotrophic nitrite oxidizers within *Chloroflexi* remains unclear, even in the environments from which they were isolated, these discoveries have provided an insight into the evolution of nitrite-oxidizing bacteria. A phototrophic origin had previously been suggested for *Nitrobacter* and *Nitrococcus* based on their cell morphology and 16S rRNA-based phylogenetic inference (117), and this hypothesis was strongly supported by the discovery of phototrophic nitrite-oxidizing bacteria in the genera *Rhodopseudomonas* and *Thiocapsa*, which are closely affiliated with *Nitrobacter* and *Nitrococcus*, respectively. Moreover, comparative genomic analysis of nitrite oxidoreductase (Nxr) loci indicated lateral gene transfer events between *Nitrolancetus* and other nitrite-oxidizing bacteria carrying cytoplasmic Nxr including *Nitrobacter* and *Nitrococcus*, which suggested that the horizontal transfer of the Nxr module allowed the spread of nitrite oxidation ability during bacterial evolution.

### 3) CH_4_-dependent denitrifying bacteria (Fig. 1B [3], Table 1)

**Fig. 2:**
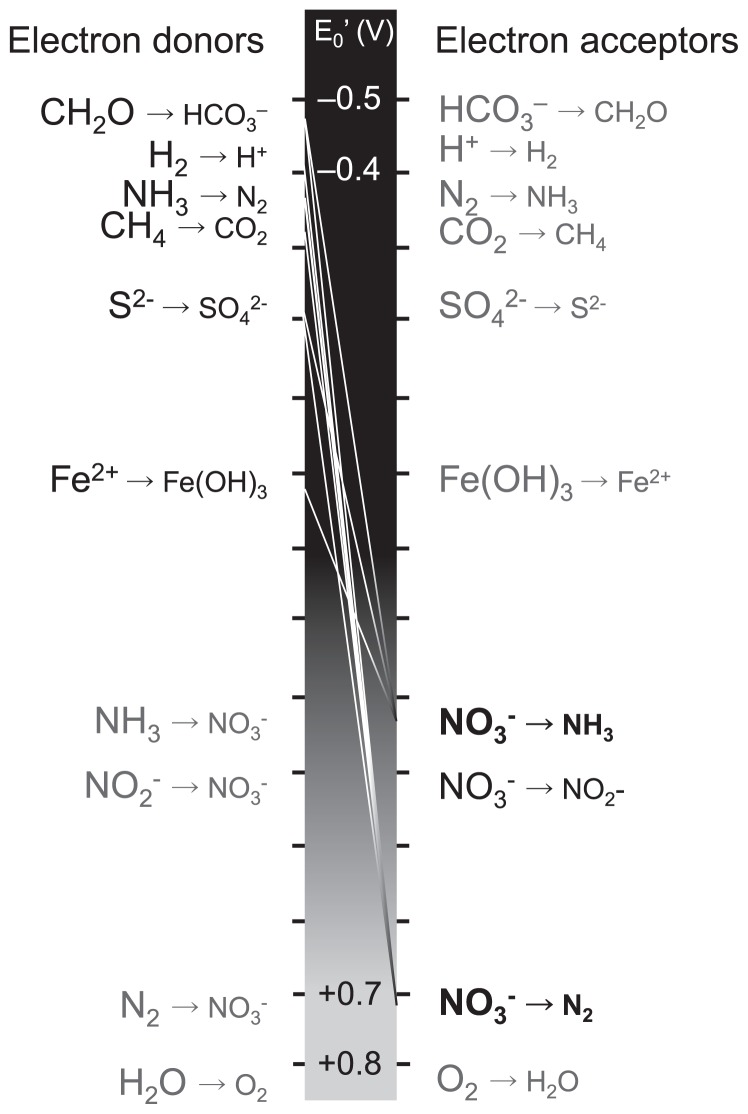
Representative reactions that have been confirmed with bacterial isolates of nitrate reduction to N_2_ or NH_3_ coupled with the oxidation of reductants ranging from organic carbon to Fe (II) along the redox tower.

Microbes that couple the formation of N_2_ with the oxidation of organic carbon (C) (organotrophic denitrification) have been examined in detail; however, some microbes can couple the formation of N_2_ with the oxidation of various reductants other than organic C (Fig. 2). The coupling reaction with CH_4_ is currently receiving attention because of its biogeochemical and evolutionary importance (23). The anaerobic oxidation of CH_4_ coupled with denitrification is thermodynamically feasible; therefore, it has been speculated that this reaction could occur in nature. Raghoebarsing *et al.* (96) provided empirical evidence from an enrichment culture from sediment in a Dutch canal, which consisted of a co- culture of a dominant bacterial phylotype of the candidate phylum NC10 and archaea that were phylogenetically positioned between *Methanosaeta* (methanogenesis) and ANME-2 (anaerobic methanotrophs), later named ANME-2d. Ettwig *et al.* (22, 23) subsequently showed that the complete anaerobic oxidation of CH_4_ coupled with the reduction of nitrite to N_2_ could be achieved using bacteria identified as “*Candidatus* Methylomirabilis oxyfera” in the absence of archaea. Genome analysis of “*Ca.* M. oxyfera” revealed that this bacterium possessed a well-established aerobic pathway for CH_4_ oxidation, whereas it lacked known genes for N_2_ production (the gene cluster encoding enzymes for the reduction of N_2_O to N_2_ [*nosZDFY*]). Isotopic labeling experiments also revealed that “*Ca.* M. oxyfera” bypassed the denitrification intermediate, N_2_O, by converting two NO molecules to N_2_ and O_2_, which was then used to oxidize CH_4_. The proposal of this metabolic pathway had a significant impact on the “evolution of organisms” debate because the suggested intra-aerobic metabolism allowed for the possibility that oxygen was available for microbial metabolism before the evolution of oxygenic photosynthesis. The metabolic mechanisms of “*Ca.* M. oxyfera” may be controversial because the presence and nature of the oxygen-producing enzyme are unknown. However, the functional importance of CH_4_-dependent denitrifiers in both natural and engineered environments has been suggested by the detection of 16S rRNA genes or *pmoA* of NC10 bacteria with high similarity to “*Ca.* M. oxyfera” in wastewater sludge (74), paddy soil (125), and oligotrophic lake sediments (63). Haroon *et al.* (35) more recently revealed that archaea belonging to ANME-2d, named “*Ca.* Methanoperedens nitroreducens”, which were co-enriched with bacteria of the candidate phylum NC10 in their consortia, exhibited CH_4_-dependent nitrate reduction to N_2_ (40) through metagenomic, single-cell genomic and metatranscriptomic analyses combined with isotopic labeling experiments. “*Candidatus* M. nitroreducens” possesses genes that reduce nitrate to nitrite (*narGH*), but lacks genes for the subsequent steps in denitrification, and can supply nitrite to “*Ca.* M. oxyfera” in the consortia by coupling nitrate reduction to nitrite with anaerobic CH_4_ oxidation in a reverse methanogenesis pathway.

### 4) Non-denitrifying N_2_O-reducing bacteria (Fig. 1B [4], Table 1)

Nitrous oxide is a greenhouse gas that is controlled under the Kyoto Protocol. Among non-CO_2_ greenhouse gasses, the contribution of N_2_O to climate forcing is second only to methane, and has a global warming potential that is ca. 300 times greater than an equivalent amount of CO_2_ (97). It has also been shown to be the single most dominant ozone-depleting substance (97). The production and consumption (reduction) of N_2_O are largely governed by microbial activities. N_2_O is produced mainly through denitrification and nitrification. Genome analysis of *Agrobacterium tumefacience* first revealed that denitrifying bacteria can lack the Nos gene, which codes for the N_2_O reductase that catalyzes the reduction of N_2_O to N_2_ (129). Approximately one-third of the genomes that possess Nir genes, which encode the nitrite reductases necessary for denitrification, are known to lack *nosZ*. Genome analysis revealed that aerobic ammonia-oxidizing bacteria and archaea also lacked *nosZ*. The reduction of N_2_O to N_2_ has been attributed to *nos*-possessing denitrifiers (94). However, recent studies demonstrated that N_2_O reducers were not always the denitrifiers. Sanford *et al.* (102) and Jones *et al.* (51) performed comprehensive phylogenetic analyses of the full-length *nosZ* in genomes retrieved from a public database and discovered that *nosZ* phylogeny formed two distinct clades (clades I and II). Clade II has not yet been detected with widely used primer sets or accounted for in studies on N_2_O-reducing communities. Jones *et al.* (51) designed primers to detect *nosZ* in clade II and showed that *nosZ* from clade II was at least as abundant as that from clade I in various environments using quantitative PCR. In addition, Sanford *et al.* (102) showed that approximately half of the genomes from phylogenetically diverse microbes containing *nosZ* from clade II (*Delta- and Epsilon-proteobacteria, Verrucomicrobia, Bacteroidetes, Chlorobi, Firmicutes, Deferribacteres*, and *Euryarchaota*) lacked *nirK* or *nirS*. They subsequently verified the physiological function of *nosZ* from clade II as an N_2_O reductase in growth experiments using the non-denitrifying species, *Anaeromixobacter dehalogenans*, which is widely and abundantly distributed on land, and N_2_O as an electron acceptor. Accordingly, these sequential studies showed that *nosZ* was more diverse than previously thought and that non-denitrifiers possessing *nosZ* were widely distributed, at least on land (51, 102). Denitrification is not always the result of successive reactions carried out in a single cell, but can result from successive reactions in microbial communities. Non-denitrifying populations with a broad range of metabolisms and habitats may be significant contributors to the mitigation of N _2_O emissions.

### 5) Aerobic ammonia-oxidizing archaea (Fig. 1 [5], Table 1)

Ammonia oxidation, the first and rate-limiting step of nitrification, was considered to be performed mostly by certain groups of chemolithoautotrophic *Proteobacteria* (genera *Nitrosospira*, *Nitrosomonas*, and *Nitrosococcus*) for more than one hundred years (44). The recent discovery of homologs of ammonia monooxygenase (Amo) genes in archaea of the phylum *Thaumarchaeota* and the cultivation of thaumarchaeal ammonia oxidizers (“*Candidatus* Nitrosopumilus maritimus”, a marine group I.1a representative; “*Candidatus* Nitrososphaera viennensis” and “*Candidatus* Nitrososphaera gargensis”, soil group I.1b representatives isolated from soil and enriched from a hot spring, respectively; “*Candidatus* Nitrosocaldus yellowstonii”, thermophilic ThAOA or HWCGIII representatives enriched from a hot spring; “*Candidatus* Nitrosotalea devanaterra”, a soil group I.1a-associated representative enriched from soil) has radically changed this view, indicating that an additional, predominant group of microbes is also able to perform this process.

Accurate estimates of nitrification activity may revise our understanding of oceanic productivity (76, 133). Nitrate is the most abundant form of fixed N in open oceans (124), and nitrification was, until recently, believed to occur almost entirely in deep waters (133) because ammonia oxidation is inhibited by light and ammonia concentrations in surface waters are generally markedly lower than those estimated to represent the growth threshold of AOB. Therefore, nitrate was thought to be a non-regenerated nutrient form in the euphotic zone, and nitrate uptake in surface waters was generally ascribed to new primary production. However, physiological studies on “*Ca.* N. maritimus” (75) have suggested that marine ammonia-oxidizing archaea (AOA) may be adapted to these low ammonia levels. Both its extremely low substrate threshold and half-saturation constant are unprecedented, but consistent with the conditions found in oligotrophic open oceans and effectively compete with bacterio- and phytoplankton. Advances in ^15^N measurement techniques have also revealed the occurrence of nitrification in the euphotic zone or at the bottom of this zone, most likely by the thaumarchaeal ammonia oxidizers (16). Accurately quantifying the role of nitrification in the production of nitrate in oligotrophic surface waters will contribute to more realistic model predictions of ocean productivity.

The successful enrichment of acidophilic “*Ca.* N. devanaterra” within thaumarchaeota group I.1a-associated has provided a new solution to the longstanding paradox of nitrification in terrestrials. Approximately 30% of the world’s soils are acidic (pH <5.5) and autotrophic ammonia oxidation can occur in acidic soils. However, all cultivated aerobic AOB readily enriched from acid soils are neutrophilic, and none grow in liquid batch-cultures with pH below 6.5 (18). Therefore, some ammonia oxidation mechanisms have been proposed in acidic soils (*e.g.*, the presence of a neutrophilic space or the ureolytic growth of AOB in soils) (18). However, ^13^CO_2_-DNA-stable-isotope probing (SIP) experiments convincingly linked the autotrophic nitrification activity in acidic soils to thaumarchaeal ammonia oxidizers (68, 141). Additionally, the growth of “*Ca.* N. devanaterra” was shown to be chemolithotrophic and optimal in a pH range between 4 and 5, unlike all previously cultivated ammonia oxidizers. Moreover, the pH selection of soil thaumarchaeal ammonia oxidizers was demonstrated using 454 barcoded pyrosequencing, which identified group I.1a-associated thaumarchaeal *amoA* lineages with specific adaptations to acidic soils (34). These studies have provided a plausible explanation for the high rates of nitrification in acidic soils and also confirmed the vital role played by thaumarchaea in meditating ammonia oxidation in acidic soils.

### 6) Anaerobic ammonia-oxidizing bacteria (Fig. 1 [6], Table 1)

The discovery of anammox (anaerobic ammonium oxidation) filled in certain knowledge gaps in the N loss pathway (19). Oceanographers previously reported a pervasive loss in ammonium in highly stratifiedanoxic basins since the mid-1960s from analyses of the N balance, which indicated that ammonium was removed by anaerobic microbial activity (99). According to a thermodynamic perspective, the physicist Broda (10) also proposed the existence of lithotrophic microbes that could derive their energy for growth from the oxidation of ammonia coupled with the reduction of nitrate or nitrite to produce N_2_. This empirical discovery was made in the bioreactors of wastewater treatment plants in the 1990s. Strous *et al.* (113) obtained a highly enriched culture of anammox bacteria, named “*Candidatus* Brocadia anammoxidans” within the order *Planctomycetales* by density gradient centrifugation. The culture produced N_2_ from ammonium and nitrite and was capable of CO_2_ fixation (86, 112, 113). Since then, five genera of anammox bacteria have been (provisionally) described: “*Brocadia*”, “*Kuenenia*”, “*Anammoxoglobus*”, “*Jettenia*” (all fresh water species), and “*Scalindua*” (marine species). The anammox reaction has been detected not only in anoxic wastewater, but also in natural environments such as marine, coastal, and estuarine sediments, anoxic basins, mangrove sediments, oceanic oxygen-depleted zones, freshwater sediments, and even in agricultural soils (3, 4, 60, 67, 69, 103, 132, 136, 142). The control of denitrification and anammox, which are the main N loss processes and fundamentally rely on different organisms and metabolic pathways, is receiving particular attention in the ocean and is discussed below.

## Relationship between microbial community dynamics and N biogeochemistry

As described above, the discovery of novel processes and players has greatly broadened our knowledge of how N is transformed and utilized in ecosystems. In this section, we have discussed attempts to elucidate the relationship between microbial community dynamics and N biogeochemistry. We also highlighted how such approaches have advanced our understanding of the biogeochemical roles of microbial communities in N cycles.

Microbiologists have attempted to identify the specific microbes responsible for N transformation processes and describe how their population dynamics impact N transformations. However, it is generally difficult to identify the biogeochemical roles of specific microbes involved in the assemblages of diverse microbial communities. Therefore, microbiologists often make rough estimates of their biogeochemical roles by correlating microbial community dynamics with environmental gradients and/or changes in N biogeochemical properties. The basic concept of linking microbial community dynamics with N biogeochemistry is based on individual processes in the N cycle being mediated by a certain group of the microbial community, with the population dynamics of this group being likely to affect the rate of the corresponding process (106). This response is most likely to be observed when the process is physiologically defined and when the responsible microbes are metabolically and phylogenetically limited. Ammonia oxidation, for example, has physiologically been defined as ammonia oxidation to nitrite via hydroxylamine. The microbes responsible are metabolically and phylogenetically limited groups (genera *Nitrosospira*, *Nitrosomonas*, and *Nitrosococcus* within the phylum *Proteobacteria* and *Candidatus* genera “*Nitrosopumilus*”, “*Nitrososphaera*”, “*Nitrosocaldus*”, and “*Nitrosotalea*” within the phylum “*Thaumarchaeota*”). The positive correlation between the quantity of *amoA* and rate of gross ammonia oxidation (nitrification) has frequently been observed in many environments such as forests, agricultural soils, and the ocean (7, 36, 46, 127), and has permitted us to identify the groups involved in ammonia oxidation (*e.g.*, proteobacterial or thaumarchaeal ammonia oxidizers). For example, Di *et al.* (21) suggested that ammonia oxidation in N-rich grassland soils was mainly driven by proteobacterial ammonia oxidizers by showing that the *amoA* quantity of *Proteobacteria* in soil was related to the net nitrification rate, whereas the *amoA* quantity of “*Thaumarchaeota*” was not. The observed correlation also allowed us to explain changes in the nitrification rates along environmental gradients by changes in the population size or community compositions of ammonia oxidizers (127). Hawkes *et al.* (36) showed that changes in the gross nitrification rate along with changes in the plant community after exotic plant invasion in grasslands could be explained by the population size of AOB. The basic concept linking microbial community dynamics with N biogeochemistry apply to the majority of other dissimilating processes in N-cycling, including N-fixation, nitrite oxidation, denitrification, N _2_O reduction, and anammox (5, 38, 43, 58, 67, 100, 135). Spatial variations in N_2_O/N_2_ emission potential in a grassland pasture can be described using spatial variations in the relative abundance of *nirS* and *nirK* in denitrifiers to *nosZ* in denitrifiers and non-denitrifies (93). Differences in the N loss pathway in oceans could be explained by the population size or community composition of denitrifiers and anammox bacteria (123). However, we need to be aware that only a rough estimate of their biogeochemical roles can be made because of limitations and biases in the basic concept. Other environmental factors (*e.g.*, resource supply) may have a greater impact on the rates of N transformations than microbial community dynamics. Microbes are generally metabolically versatile; therefore, population dynamics do not always cause changes in the specific process rate. We have generally focused on sensitive microbial groups, the population size of which may change, with less attention been given to the functional importance of insensitive groups. Additionally, there may be a discrepancy between the presence of a gene (or even mRNA) and *in situ* activity. Therefore, RNA- or protein-based analyses such as metatranscriptomic or proteomic analyses combined with substrate uptake assays such as SIP, microautoradiography combined with fluorescence *in situ* hybridization (MAR-FISH), and NanoSIMS are powerful tools that can be used to refine rough estimates. The series of attempts to link microbiological community dynamics with N biogeochemistry discussed here have provided a more mechanistic understanding of N dynamics than a direct observation. These findings should also contribute to more realistic model predictions of the N cycle.

## Highlighted Topics

We here highlighted three topics that have not been examined in detail, but are essential for identifying the roles of microbial community dynamics in the functional stability of the ecosystem. These topics are fundamental for understanding the control of ecosystem functioning, including (1) how bioavailable N is microbially supplied, (2) how the N supplied is dissimilatorily transformed to yield energy, and (3) how the storage of N in an ecosystem through microbial N assimilation using energy contributes to the stability of the ecosystem.

### 1) N mineralization and the supply of available N (Fig. 3 [1])

The first topic discussed is the mineralization of N-containing organic compounds to NH_4_^+^ and the microbes responsible for this transformation. The mineralization of dissolved organic N (DON) to NH_4_^+^, which represents a bottleneck in the subsequent N-cycling processes and the supply of available N to organisms in various environments, occurs through microbial enzymatic activity. However, little progress has been made in understanding the relationship between N mineralization and microbial community dynamics. The main reason for this may be that the basic concept linking microbial community dynamics with N biogeochemistry discussed above cannot be sufficiently applied to N mineralization (106). We generally measure N mineralization in the form of ammonium production as a single process; however, it is actually the sum of multiple distinct physiological processes. Therefore, N mineralization can involve diverse microbial communities that contribute to the process; N mineralization rate may be insensitive to the dynamics of the microbial community involved in the mineralization (106).

**Fig. 3:**
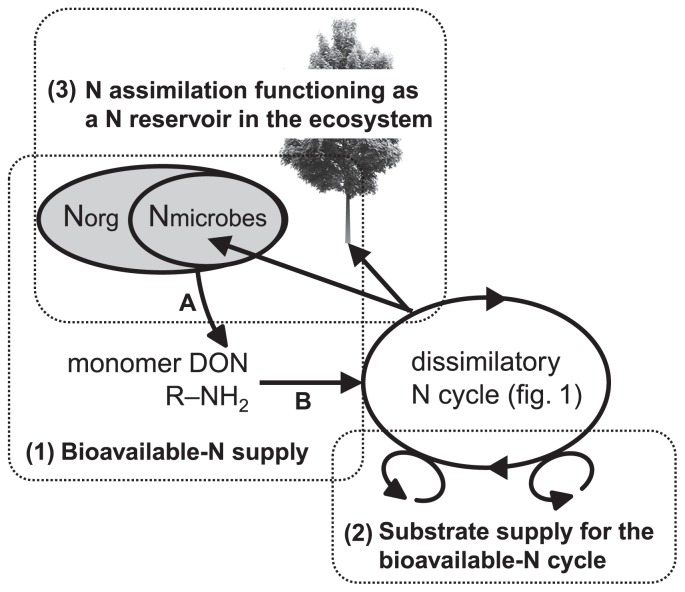
Schematic outline of N flows and microbial involvements at the ecosystem level, as discussed in this review. (1) bioavailable N is microbially supplied through the mineralization of organic N including microbe-derived N, (2) supplied bioavailable N is dissimilatorily transformed in the redox reaction with other oxidants/reductants to yield energy, and (3) the storage of N in an ecosystem through microbial N assimilation using this energy contributes to the stability of the ecosystem. A: depolymerization; B: ammonification.

Biogeochemists are currently attempting to break down the entire process of mineralization into individual processes, which may then be physiologically defined and more sensitive to microbial community dynamics. Schimel and Bennet (105) suggested that a single mineralization step in soil should be separated into at least two processes that are under different microbial control, namely, depolymerization (proteolysis and aminization; organic-N polymers to R-NH_2_) (Fig. 3 [A]) and ammonification (deamination and deamidation; R-NH_2_ to NH_3_ + H_2_O) (Fig. 3 [B]). Ocean biogeochemists have agreed that this fractionation should be performed to enable N regenerated production to be accurately described (54, 80). Ammonium can potentially be produced by the direct enzymatic cleavage of a free amino group, either amine- or amide-N (R-NH_2_). Because deaminase and deamidase enzymes are intercellular in nature, are active inside living organisms, and can be produced by most microbes, the microbial production of NH_4_^+^ can depend on the N status of cells taking up small organic N compounds (R-NH_2_), which will subsequently determine if N is sequestered or excreted as NH_4_^+^. Small organic N monomers such as amino acids, amino sugars, and nucleotides are produced by the activities of several extracellular enzymes, which break down high-molecular-weight polymeric organic compounds, including proteins, cell wall polymers (aminopolysaccharides), and nucleic acids. For example, proteins are broken down by a wide variety of proteinases and peptidases. Proteinases break down large proteins, while peptidases may cleave tri- or dipeptides or split off an individual amino acid, which is then taken up by a microbe. Amino-polysaccharides are also broken down by extracellular enzymes. Chitinase depolymerizes chitin (a polymer of *N*-acetylglucosamine), which forms the cell walls of many fungi and is also a part of insect exoskeletons. Chitinase breaks chitin into dimers of chitobiose. *N*-acetylglucosaminidase subsequently cleaves chitobiose into two molecules of *N*-acetylglucosamine. Several enzymes degrade the peptidoglycan portion of bacterial cell walls. For example, lysozyme breaks the β-1,4 linkage between *N*-acetylmuramic acid and *N*-acetylglucosamine. The end products of extracellular enzymes that degrade microbial cell walls are individual amino sugars, which are taken up by microbes. Because each of the extracellular enzymes used in proteolysis and aminization may be synthesized by a more limited group of microbes than the intercellular enzymes used in deamination and deamidation, this process may become more physiologically defined and, thus, more sensitive to the dynamics of the microbial community involved in the extra-cellular enzyme steps.

Microbiologists are currently attempting to identify the microbial groups that contribute to the extracellular enzyme steps in order to link this process with microbial community dynamics. Depolymerization occurs, to some degree, in most environments; therefore, if this process is carried out by specialized or phylogenetically limited microbial groups, such groups may be ubiquitous. Zimmerman *et al.* (143) recently analyzed 3,058 annotated prokaryotic genomes to identify taxa with the genetic potential to produce chitinase and *N*-acetylglucosaminidase, and found a non-random correlation between genetic potential and 16S rRNA-based phylogeny. Chitinase- and *N*-acetylglucosaminidase-positive genotypes were detected in 12 and 19 of the 30 phyla. All genomes of the phylum *Acidobacteria*, which are ubiquitous and abundant in soil, but have unknown ecological characteristics (49, 52), possessed both chitinase and N-acetylglucosaminidase-encoding genes, which allowed the complete hydrolysis of chitin substrates by individual organisms. Most of the genomes of the genus *Vibrio*, which can be found in a range of aquatic habitats, also possess these genes. These ubiquitous groups may be actively involved in the depolymerization process in terrestrial or aquatic environments. Identifying and studying the ecology of the microbial communities responsible for N mineralization combined with enzyme activity analyses and proteomics will provide a deeper insight into how the supply of bioavailable N can be microbially controlled to maintain the functional stability of ecosystems.

### 2) N-dissimilating reactions and substrate availability/limitation (Fig. 3 [2])

The second topic relates to the supply of substrates for dissimilating N reactions. Previous studies on dissimilating N transformations have primarily focused on the flow of N. However, other substrates in N transformation redox reactions that are reduced when N is oxidized (or oxidized when N is reduced) are also regulating factors that drive the flow of N. The functioning of an ecosystem can primitively be supported by the energy yielded by microbial N-dissimilating reactions because each microbial function can be maintained by this energy, and the sum of these functions may represent the principal functions of the ecosystem. Therefore, the nature of available, but limited substrates that drive energy-yielding N transformations can characterize ecosystems. For example, we may estimate the denitrifier’s control on the denitrification rate in an ecosystem by tracking the genes of denitrifiers (such as *nirS*, *nirK*, and *nosZ*) and N-gas emissions, as discussed in the previous section. However, the nature of available substrates other than nitrate may have a stronger influence on denitrification rates by altering denitrifying microbial communities. Moreover, the diversification of the substrate that microbial communities in ecosystems utilize for energy-yielding N transformations is strongly relevant to the robustness of the ecosystem.

Many pairs of electron donors and acceptors have been observed in each N dissimilation reaction using enrichment cultures or isolates. The recent findings described above have also revealed new electron donors/acceptors and energy sources (phototrophic nitrite-oxidizing bacteria, CH_4_-dependent denitrifying bacteria, non-denitrifying N_2_O reducing bacteria, and anaerobic AOB) for N transformations. The microbial reduction of nitrate, for example, is often considered to be coupled with the oxidation of organic-C (*e.g.* acetate, succinate, and glucose) on land; however, this reduction coupled with the oxidation of reductants ranging from organic C to Fe (II) along the redox tower has been reported previously in bacterial isolates (11) (Fig. 2). The constituents of organic C may be selectively used for microbial growth. Additionally, reactions observed in a single cell can also be performed in the surrounding cells if there is a flow of electrons between these cells (57, 98).

The spatial distribution and ecological importance of these nitrate-reducing reactions coupled with reductants other than organic carbon have not been sufficiently studied at the ecosystem level. However, several recent studies have suggested that the supply of available substrates could regulate the pathway of N loss through nitrate reduction in an ecosystem. Kuyper’s group reported that N was lost in the Black Sea suboxic zone and Peruvian oxygen-minimum zones (OMZs), which mainly occurred through an ammonia-dependent denitrification (anammox) reaction, and also that anammox was coupled with an ammonia production process known as dissimilatory nitrate reduction to ammonium (DNRA) (Fig. 1A) (67) or DON ammonification (55) using ^15^N-labelling experiments corroborated by functional gene expression analyses. On the other hand, Ward’s group showed that N was lost in the OMZs in the Arabian Sea mainly via DOC-dependent denitrification under high DOC production conditions (126). Dalsgaad *et al.* (17) more recently showed that both anammox and denitrification were detected in a transect along the coast of South America (the eastern tropical South Pacific OMZ), and that anammox occurred at low rates at almost every station, whereas denitrification was less commonly detected, but occurred at very high rates at a few stations. These findings suggest that the timing and magnitude of the DOC supply may regulate the relative contribution of anammox and denitrification in the anoxic region of oceans. Regarding the other mechanisms, Hayakawa *et al.* (37) revealed from the stoichiometric analysis of sulfur and nitrogen that N was lost through sulfur-dependent denitrification in FeS-rich sediments. Yang *et al.* (131) proposed a new N loss pathway from tropical upland soils, anaerobic ammonium oxidation to N_2_ coupled with iron (III) reduction, otherwise known as Feammox, based on the results of their ^15^N-labeling experiments. Although this reaction can be thermodynamically favorable (Fig. 2), the microbes that could govern this reaction have not yet been identified and the possibility of a corporative reaction with organotrophic denitrification should not be ruled out. As described here, studies on new electron donors or acceptors in N loss reactions as well as the importance of new N loss pathways are being conducted at the ecosystem level (Fig. 2). Further studies on the diversity and variability of the substrates utilized in N transformations are needed. Understanding the diversity and variability of substrates and related microbial community dynamics may allow us to understand the nature of the available, but limiting resources of the ecosystem and also characterize ecosystems.

### 3) N-assimilating reactions and their functions as a N reservoir (Fig. 3 [3])

Ecologists have historically considered that communities of organisms are primarily structured by available resources and, in particular, by the nature of the limiting resource (9). A variant in the substrate partitioning theory, which was built around the Monod model of microbial growth, has effectively been used to explain the microbial community dynamics (9, 128). The properties associated with substrate partitioning have been expressed by the terms copiotrophy and oligotrophy in microbiology (r-strategy and K-strategy in ecology, respectively) (26, 62). These terms have primarily been used to describe the relationship between growth rates and carbon concentrations (typically in the medium in cultivation) (62). Fierer *et al.* (26) showed that growth responses to sucrose amendments in soils slightly differed among taxa at the phylum/class level (phylum *Acidobacteria* and *Bacteroidetes*, class *Betaproteobacteria*). Martiny *et al.* (77) analyzed the distribution of 89 functional traits, most of which were associated with simple carbon utilization, across a broad range of prokaryotes using genome and phenotypic carbon substrate utilization data, and found that the capacity for simple carbon utilization correlated with the 16S rRNA-based phylogeny. However, carbon is not always the limiting substrate for microbial growth. Previous studies have shown that many natural environments, including terrestrial areas, lakes, and oceans, are frequently N-limited. Microbial community dynamics may be reflected in both the nature of the carbon sources and supply of N available for their growth in such environments. However, few studies have explored the N assimilation kinetics of each microbial taxon in such environments and the link between N assimilation properties and microbial community dynamics has not been elucidated in detail (30, 61). Taxon-specific mechanisms are difficult to isolate because all microbes are involved in the N assimilation process. Furthermore, measuring the N assimilation rate is methodologically difficult (particularly in the case of soils) because of the difficulty involved in separating microbial cells from soil particles (45). Therefore, we can describe community dynamics, but do not have an appropriate theory or model to explain these dynamics in N-limiting environments.

Another important aspect of N assimilation by microbial communities and microbial growth is their function as an N reservoir in an ecosystem. Microbial biomass N is the main component of organic N in an ecosystem and can, thus, be a source of bioavailable N. The lysis of microbial cells and subsequent release of N (depolymerization and ammonification) can supply bioavailable N. For example, we emphasized the importance of microbial N assimilation and community dynamics in forest ecosystems. Plants in many boreal and temperate forests strongly demand N for their growth and increased productivity in late spring and summer. Therefore, the supply of N available to plants must be greater during the growing season. Bacterial growth and an increase in the biomass N in summer and autumn and bacterial death and subsequent release of biomass N in winter may be important for meeting the N demands of plants in the coming spring and summer. The activity of the bacterial community in soil has generally been correlated with the soil temperature; thus, it is low in winter. Seasonal variations have been reported in bacterial communities, with the bacterial population size being smaller in the winter. Furthermore, bacterial death can be accelerated when soils are frozen and thawed repeatedly. Therefore, a large quantity of N from bacterial biomass can be released into the soil in the winter. The degree to which bacteria-derived organic N can be utilized by plants in the plant-growing season remains unclear. However, Shibata *et al.* (109) recently showed that increases in freeze-thaw cycles, which accelerate bacterial death and the physical degradation of organic N, significantly enhanced the net production of ammonium in soil during the winter, which indicated that bacteria-derived organic N may be an important source of NH_4_^+^. Litters that fall in winter and fine roots that are killed in winter provide additional N. The depolymerization and/or ammonification of bacteria-derived organic N can be accelerated by increases in temperature or snow melts in the spring, but may also occur in soil in the winter, particularly when it is not frozen. The Schmidt group (71, 72, 104) used light microscopy to show that fungal biomass reached its annual peak with high diversity under snow in tundra soils, although their function in the mineralization of bacteria-derived organic N remains unknown. To date, bacterial growth and metabolism have been confirmed at −15°C and −32°C, respectively (6, 87). Understanding 1) the microbial community dynamics involved in bacterial growth before winter, 2) bacterial death and mineralization of the released N in winter, and 3) the competition with plants for N assimilation in the plant-growing season will allow us to identify the role of microbial community dynamics in the functional stability of the forest ecosystem.

Regional variations in the seasonal succession of the roles of microbial community dynamics in the functional stability of an ecosystem should not be ignored. For example, forests located in the monsoon climate region in Japan generally have the highest water discharge rate during the plant-growing season (early summer) because precipitation inputs are typically high (89). The high discharge rates during this season are in marked contrast to forests in most regions of the United States and Europe, in which the summer period is characterized by high evapotranspiration, but also by low or moderate precipitation inputs that result in low water discharge rates (81, 89). High water discharge causes the marked loss of nitrate, which is the main source of available N for plants, through leaching from the soil. Thus, the magnitude and timing of the mineralization of microbe-derived organic N and subsequent nitrification may be more strictly regulated based on the season in forests in Japan than in the United States and Europe. Studies that can evaluate such regional differences in the ecological consequences of microbial community dynamics will allow us to characterize regional variations in ecosystems and should also provide a deeper insight into how ecosystems will respond to environmental changes.

## Concluding remarks

We have presented three topics that could further our understanding on the role of microbial community dynamics in the functional stability of ecosystems. These topics are not limited to functionally equivalent microbial groups or to specific (a single or few) N transformation processes. Therefore, the basic concepts that are discussed in the previous section and used to link microbial community dynamics with N biogeochemistry are not pertinent. Moreover, advances cannot be expected simply by using high-throughput sequencing techniques to examine the assembly of individual genes, even though these techniques have led to great successes in the heuristic search for gene diversity. Substrates that drive N transformations, the energy efficiency of N transformation redox reactions, competition for available N, and mutual interactions between micro- and macro-organisms must also be considered. These kinds of studies by microbiologists can potentially provide a more mechanistic understanding of the fundamental issues of ecosystem ecology in unprecedented levels of detail.

Another topic that ecosystem ecologists have focused on is how a diverse range of organisms can contribute to the resilience of an ecosystem (2, 31). Phylogenetically limited ammonia oxidizers are known to be responsible for critical functions in an ecosystem (nitrification). The ammonia oxidation rate is more likely to be influenced by environmental changes or ecosystem perturbations than the mineralization rate (32, 92), which may be attributed to the markedly lower phylogenetic diversity of ammonia oxidizers than that of microbial communities involved in mineralization. Thus, the associations between microbial diversity and stability of the biogeochemical functions of the microbial community may directly impact the resilience of an ecosystem.

Recent technological advances have permitted microbiologists to assess these topics in ecosystem ecology. Advances in high-throughput sequencing have allowed microbiologists to better delineate microbial phylogenetic and functional diversities through meta-genomic, transcriptomic, and proteomic analyses. The use of ^15^N can also improve our understanding on microbial functional metabolism and diversity as well as ecosystem functions through Nano-SIMS, SIP, and N isotope tracer techniques. As described here, there are many fields for microbiologists to be involved in in ecosystem ecology, and they can now play indispensable roles in the fundamental issues of ecosystem ecology in collaborations with ecologists, geochemists, and geologists.

## Figures and Tables

**Table 1:** Recently characterized N cycling pathways and representatives of relevant organisms, as discussed in this review

Process	Reaction	Environments	Representative organisms	Refs*
Unicellular cyanobacterial N_2_ fixation	N_2_+ 8H^+^ + 8e^−^ + 16ATP → 2NH_3_ + H_2_ + 16ADP + 16Pi	ocean	Uncultured cyanobacteria (UCYN-A group) *Crocosphaera watsonii* (UCYN-B group)	83, 119, 120, 138, 140
Anaerobic phototrophic nitrite oxidation	NO_2_^−^ + H_2_O → NO_3_^−^ + 2H^+^ + 2e^−^	sediment/sewage	*Rhodopseudomonas* sp. (class *Alphaproteobacteria*) *Thiocapsa* sp. (class *Gammaproteobacteria*)	33, 108
CH_4_-dependent denitrification	4NO_3_^−^ + CH_4_ → 4NO_2_^−^ + CO_2_ + 2H_2_O 8NO_2_^−^ + 3CH_4_ + 8H^+^ → 4N_2_ + 3CO_2_ + 10H_2_O	soil/sediment/ wastewater sludge	“Methanoperedens nitroreducens” (phylum *Euryarchaeota*) “Methylomirabilis oxyfera” (phylum “NC10”)	23, 35, 96
Non-denitrifying N_2_O reduction	N_2_O + 2H^+^ + 2e^−^ → N_2_+ H_2_O	soil	*Anaeromyxobacter* sp. (class *Deltaproteobacteria*)	51, 102
Aerobic archaeal NH_3_ oxidation	NH_3_+ O_2_ + 2H^+^ + 2e^−^ → NH_2_OH + H_2_O NH_2_OH + H_2_O → HNO_2_ + 4H^+^ + 4e^−^	soil/ocean/lake/ sediment/ hot spring/ wastewater sludge	“Nitrosopumilus maritimus”/ “Nitrososphaera viennensis”/ “N. gargensis”/ “Nitrosocaldus yellowstonii”/ “Nitrosotalea devanaterra” (phylum *Thaumarchaeota*)	34, 65, 68, 75, 141
Anaerobic NH_3_-oxidation (nitrite-dependent NH_3_ oxidation)	NO_2_^−^ + NH_4_^+^ → N_2_ + 2H_2_O NO_2_^−^ + 2H^+^ + e^−^ → NO + H_2_O NO + NH_4_^+^ + 2H^+^ + 3e^−^ → N_2_H_4_ + H_2_O N_2_H_4_ → N_2_ + 4H^+^ + 4e^−^	soil/ocean/lake/ sediment/ wastewater sludge	“Brocadia anammoxidans”/ “B. fulgida”/“B. sinica”/ “Kuenenia stuttgartiensis”/ “Jettenia asiatica”/ “Anammoxoglobus propionicus”/ “Scalindua brodae”/“S. sorokinii”/ “S. wagneri”/“S. profunda” (phylum *Planctomycetes*)	56, 112, 113

* Other references are described in the text.
